# Asymptomatic Uterine Rupture at 20 Weeks of Gestation: A Case Report and Review of Literature

**DOI:** 10.1155/crog/2515403

**Published:** 2026-04-07

**Authors:** Marita Saliba, Barbara Jreij, Bachar Chebib

**Affiliations:** ^1^ Department of Obstetrics and Gynecology, University of Balamand, El Koura, Lebanon, balamand.edu.lb; ^2^ Department of Obstetrics and Gynecology, Haykel Hospital, Ras Maska, El-Koura, Lebanon

**Keywords:** cesarean section, intrauterine fetal demise, laparotomy, rupture, uterine rupture

## Abstract

**Introduction:**

Second‐trimester ruptures are mainly associated with trauma, induced pregnancy terminations in scarred uterus, or complications. Risk of uterine rupture is specifically increased with the use of prostaglandin, notably Misoprostol, for the induction of labor in women with a history of cesarean section and uterine scar.

**Presentation of Case:**

A 32‐year‐old female patient at 20 weeks and 3 days of gestation and intrauterine fetal demise (IUFD), with a history of C‐sections, presented to our care initially for administration of Misoprostol for management of IUFD, and was sent home on Misoprostol due to slow response at the hospital. Two days later, the patient was diagnosed with uterine rupture on ultrasound during follow‐up in the clinic. The patient, asymptomatic, was sent to the hospital for an emergency laparotomy.

**Conclusion:**

Second‐trimester ruptures are a rare occurrence but are mainly seen in patients with induction of labor with prostaglandins in the case of IUFD or abortions. Our patient had a second‐trimester rupture with no associated symptoms, which raises concern about diagnosing these ruptures before instability happens.

## 1. Introduction

Uterine rupture poses a significant risk to the mother and fetus. It mostly occurs in the third trimester of pregnancy, and earlier occurrences are exceptionally rare [1]. The incidence of uterine rupture is 5.1 per 10,000 in scarred uteri [2]. Second‐trimester ruptures are mainly associated with trauma, induced pregnancy terminations in scarred uterus or complications, such as placenta accreta spectrum [3]. Overall, 80% of uterine ruptures occur between 28 and 36 weeks of gestation [4]. The risk of uterine rupture is specifically increased with the use of prostaglandin, notably Misoprostol, for the induction of labor at term in women with a history of cesarean section and uterine scars [5]. Misoprostol is used for the induction of labor in the case of second‐trimester abortions and intrauterine fetal demise (IUFD) [6]. Other risk factors include previous surgery, including vertical hysterotomy and labor.

We present the case of a female patient who experienced uterine rupture after induction for abortion at 20 weeks of gestation, with no typical symptoms.

The work has been reported in line with the CARE guidelines.

## 2. Presentation of Case

We report the case of a 32‐year‐old female patient, G5P3A1 at 20 weeks and 3 days of gestation, who presented initially for the management of IUFD, with a history of three prior cesarean sections, and was eventually diagnosed with uterine rupture within the course of treatment.

**Figure 1:**
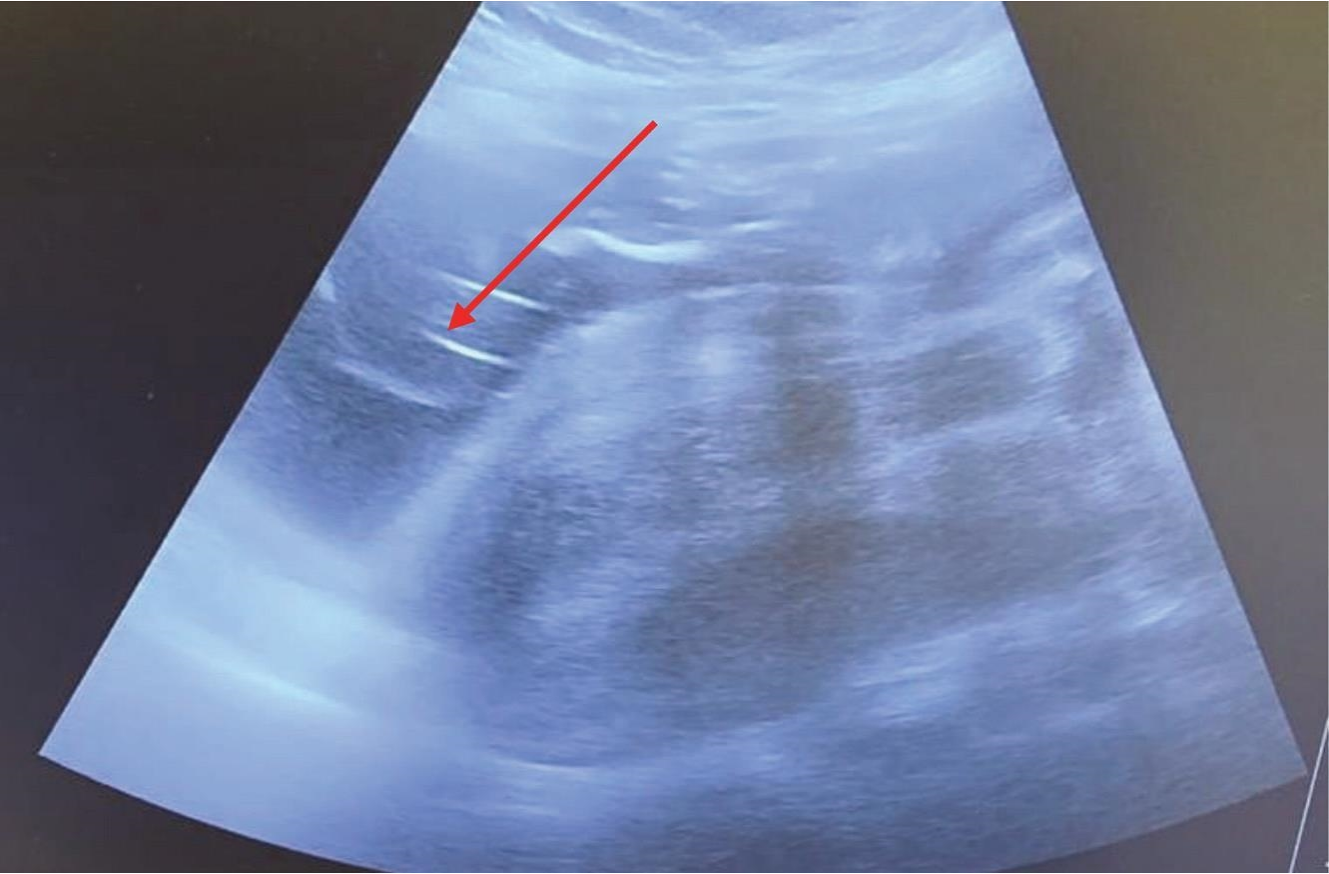
Abdominal US showing uterine rupture with amniotic sac noted outside the uterine cavity.

**Figure 2:**
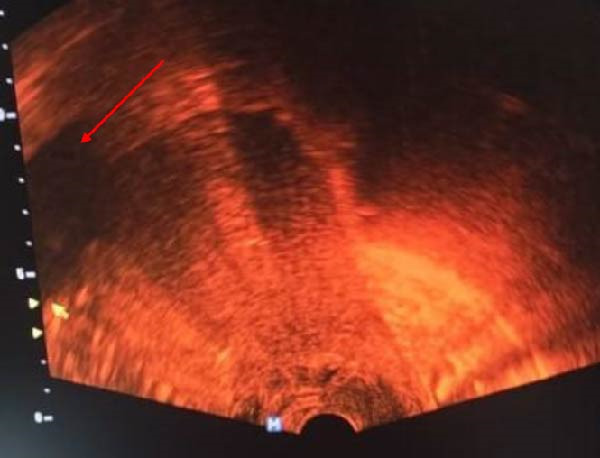
Endovaginal US showing empty uterine cavity, and amniotic sac outside the cavity (red arrow).

The patient had an uncomplicated pregnancy but was diagnosed with IUFD at 20 weeks of gestation, as no fetal heartbeat was detected on routine obstetrical ultrasound. The patient was initially admitted to our care for induction of labor, with the administration of Misoprostol 200 µg orally and 200 µg vaginally every 6 h, under surveillance and repetitive ultrasounds for assessment and management. Due to the very slow response to Misoprostol with no significant improvement, and after spending 48 h hospitalized and patient preference and request due to financial situation, she was sent home on Misoprostol 200 µg orally every 8 h to ensure safe cervical dilation at home, and eventual delivery of the demised fetus under professional supervision at the hospital. To note that the absolute risk of uterine rupture in the second trimester is substantially lower than at term because the lower uterine segment is not yet fully developed and contractile forces are generally less intense, hence the decision to switch to the frequency of a misoprostol tablet every 8 h at home after careful discussion with the patient. She was scheduled for an appointment at the clinic 2 days later to assess for improvements on Misoprostol, when uterine rupture was noted on ultrasound, with the amniotic sac herniating outside the uterine cavity through the scar (Figures 1 and 2). The patient was sent to the hospital for an emergency laparotomy. Upon arrival, she was asymptomatic with no bleeding, and the vitals were stable. Physical examination was significant for very mild tenderness only on palpation, with no pain reported. As for laboratory values, hemoglobin was 9.9 g/dL, hematocrit of 33.3%, platelets of 214 x 10^3^/µL and PT, PTT and INR of normal range. The patient was transferred to the operating room immediately after initial assessment.

**Figure 3:**
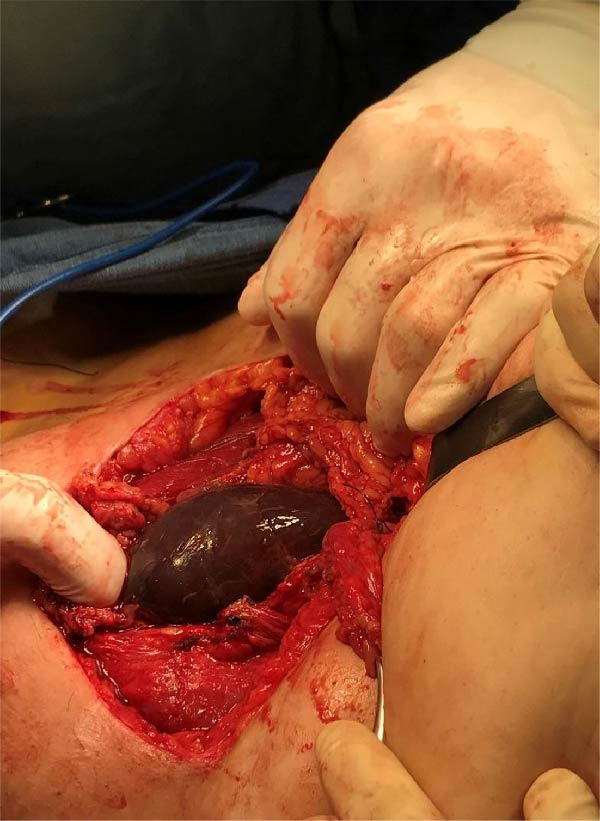
Membranes, after dissection of the rectus abdominis.

**Figure 4:**
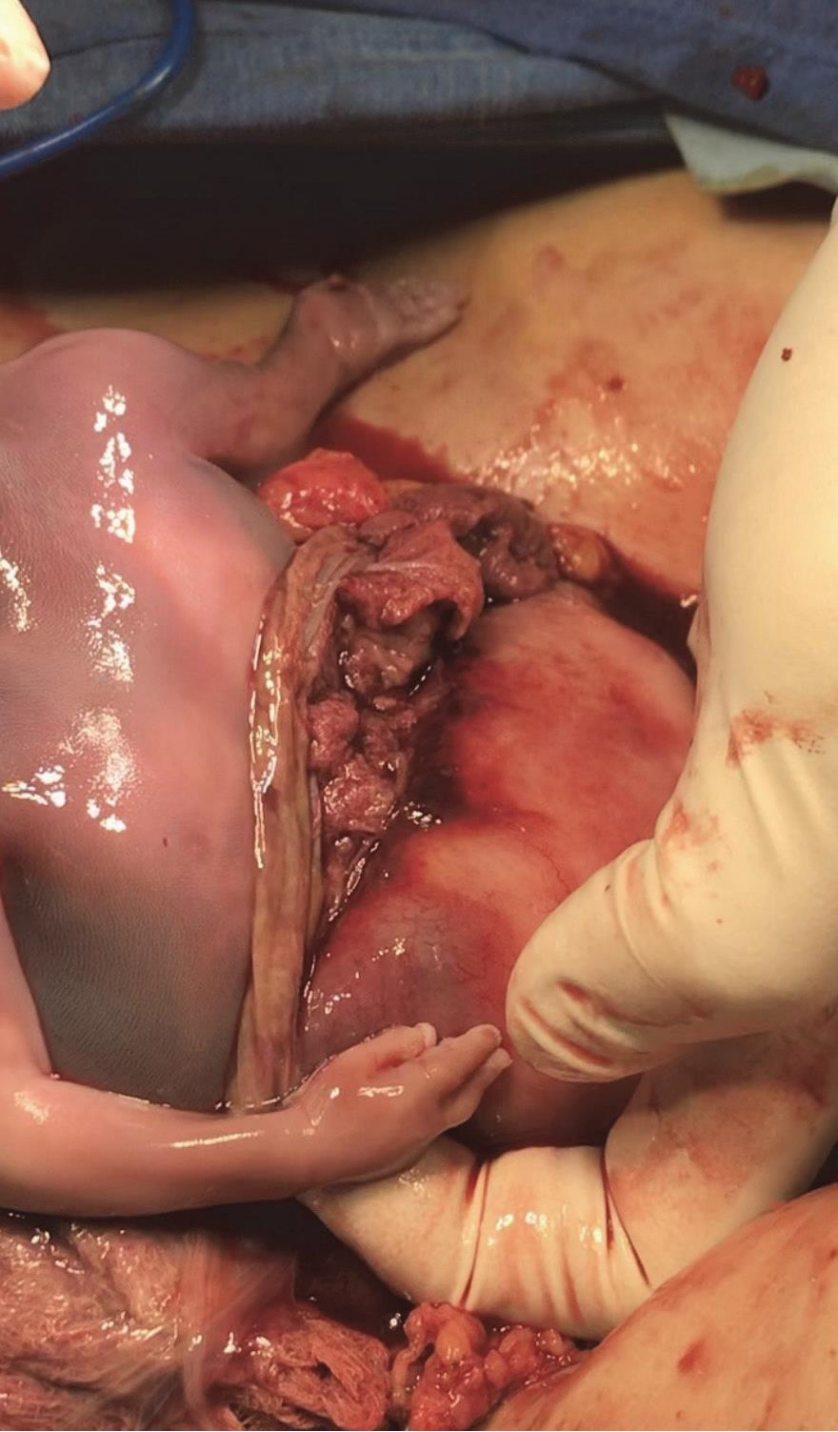
Uterine rupture site at previous C‐section scar, with fetal tissue.

Under spinal anesthesia, the patient was placed in the supine position. Pfannenstiel incision was made with entry into the abdomen layer by layer until reaching the rectus muscles. Delicate dissection was done due to heavy and thick adhesions, with hemostasis ensured after adhesiolysis. No blood or fluid was noted in the abdominal cavity due to contained rupture and unusual heavy adhesions with a frozen pelvis. Upon opening of the muscles, the amniotic sac containing the fetus was found (Figure 3). The extraction of the fetus and placenta was done, and rupture of the previous lower uterine segment cesarean scar was clearly demarcated (Figure 4). Exploration and identification of pelvic anatomy were performed. No injuries were noted.

Complete repair of the uterine rupture site was done with vicryl 1.0, and hemostasis was secured. Closure of the layers one by one was done. There was no significant bleeding intraoperatively.

The patient was then transferred to the regular floor.

The rest of her hospital stay was uncomplicated. Laboratory values were normal. The patient passed gas, urine was clear and positive after removal of the Foley catheter, and vitals were stable. She was discharged on Day‐2 postlaparotomy.

Her follow‐up examination at the clinic after 40 days was insignificant. Future plans of conception were discussed, mentioning that pregnancy would be considered high risk and require specialized care and close monitoring.

## 3. Discussion

A history of previous cesarean section is the most common risk factor for uterine rupture. Other factors include multiparity, advanced maternal age, endometriosis, placenta previa, history of dilation and curettage, myomectomy and irradiation [7, 8].

Literature has shown that the use of Misoprostol for abortions in patients with previous scarred uterus can result in rupture [9], not exceeding 1%.

A systematic review conducted by Goyal [9] analyzed the use of misoprostol and the incidence of uterine rupture. Most regimens of the studied articles included doses of 200–400 µg for women with a history of cesarean delivery, with routes of administration varying between vaginal, oral, and sublingual. The dosing intervals varied from every 3 to up to every 12 h. The risk of uterine rupture in women with a history of cesarean section was 0.28%.

Rupture was also reported with the use of natural prostaglandins PGF2 [10] and with the prostaglandin analogs gemeprost [11] and 15 methyl PGF2 [12].

The clinical manifestations of uterine rupture involve intense, severe pelvic pain, metrorrhagia and hemodynamic instability that can eventually progress to shock [13]. The case we presented did not have these typical features; she was stable with no symptoms.

Ultrasound can diagnose the site of uterine rupture as a hypoechoic or anechoic myometrial defect that extends to the serosa of the uterus. It can also show an extrauterine hematoma or sac. We can also identify air droplets, which appear as echogenic foci along the perforation tract [14].

Computed tomography (CT) is more sensitive to showing pneumoperitonem. A hypoattenuating defect and loss of myometrial continuity may be seen at the site of uterine rupture, with or without associated extra‐ or intrauterine fluid collections [14].

## 4. Conclusion

Uterine rupture typically occurs in the third trimester, in multiparas with a history of a cesarean section. Second‐trimester ruptures are a rare occurrence but are mainly seen in patients with induction of labor with prostaglandins in the case of IUFD or abortions. Uterine ruptures are associated with severe pelvic and abdominal pain and hemodynamic instability. Our patient had a second‐trimester rupture with no associated symptoms, which raises concern about diagnosing these ruptures before instability happens.

## Author Contributions

**Marita Saliba and Barbara Jreij:** writing of original and final draft. **Bachar Chebib:** supervision, reviewing and editing of the final draft.

## Funding

The authors have nothing to report.

## Consent

Informed consent was obtained from the patient for publication and any accompanying images.

## Conflicts of Interest

The authors declare no conflicts of interest.

## Data Availability

Data sharing is not applicable to this article as no datasets were generated or analyzed during the current study.
